# In Memoriam

**Published:** 1880-01

**Authors:** John G. Westmoreland, J. H. Redding

**Affiliations:** Chrm’n; Sec’y


### IN MEMORIAM.

"The great has fallen." Dr. John T. Banks is no more. At the age of about fifty, he breathed his last on Sunday morning, the fourth of January.

For twenty-five years, he was an intimate friend and associate of the writer, and from a personal knowledge of him, we are enabled to say that in his death the community in which he lived lost one of its most useful citizens, and the medical profession one of its most talented, practical members and brightest ornaments. In the general practice of medicine, including treatment of all the ills and accidents to which flesh is heir, he was deservedly eminent. High toned, honorable and just, his character stands as a model for young and rising members of the medical profession.

In his domestic relations he was exceedingly kind and tender, but firm and conscientious in the discharge of his duties as husband and father. Without deception, intrigue or prevarication, he could always be relied on as a true and uncompromising friend socially, and in business transactions, always directed by a high sense of honor and justice.

His death causes a sad loss to his family, the community in which he lived, the medical profession and the Atlanta Medical College. At great personal sacrifice he has, for several years, filled the Chair of Practice of Medicine, coming from his home in Griffin, twice a week, for the purpose. The Faculty and students highly appreciate this devotion to the cause of science, and feel keenly the pang of grief caused by their final separation from him.

We give place, below, to their expression of sorrow on this sad occasion.

ATLANTA MEDICAL COLLEGE,
ATLANTA, January 5, 1880.

The students of Atlanta Medical College met this day to pay the last tribute of respect to their late beloved Professor of the Practice of Medicine, John T. Banks, M.D.

On motion of J. A. Hurt, of Alabama, the meeting was organized by calling Prof. J. G. Westmoreland to the chair.

On motion, J. H. Redding was requested to act as Secretary.

The chairman made some pertinent remarks touching the death of his respected and beloved associate and friend, when, on motion of a committee was appointed by the chair to draft suitable resolutions expressive of the feelings of the class on the occasion of their sad loss. The committee consisted of five with the addition of the Secretary, as follows:

J. H. Holton, Ohio; James Blassdale, Maine; J. A.

Hurt, Alabama; W. R. Hollingsworth, South Carolina; W. J. Hannah, Florida; J. H. Redding, Texas.

The committee reported the following preamble and resolutions, which were unanimously adopted by a rising vote:

WHEREAS, In the dispensation of God, and the ordinary course of nature, we have been deprived of our beloved Professor of the Practice of Medicine, Dr. John T. Banks; therefore,

*Resolved*, That in his death we have sustained an irreparable loss, not only in the teaching of his important branch of medical science, but in our social relation with him as a personal friend and counsellor. Our deep grief is palliated only by the reflection that a laborious worker in the cause of science and humanity has gone to rest from the incessant turmoil of life, where watching, care, labor and responsibility are at an end.

*Resolved*, That we heartily condole with his bereaved family, and request the Secretary to forward to them a copy of our transactions, and the expression of our deep sympathy with them in their sad loss.

*Resolved*, That a copy of these resolutions be inserted in the Atlanta *Post*, Griffin daily *News* and Atlanta MEDICAL JOURNAL.

JOHN G. WESTMORELAND, Chrm'n.

J. H. REDDING, Sec'y.

